# High Fasting Blood Sugar and Increased Waist Circumference as Risk Factors for Diabetic Retinopathy in Type 2 Diabetes Patients Older than 45 Years

**DOI:** 10.7759/cureus.28291

**Published:** 2022-08-23

**Authors:** Rangabashyam Seetharaman Ranganathan, Ezhil Vendhan K, Shanmugasundaram R, Nivya Manimozhian

**Affiliations:** 1 General Medicine, Vinayaka Mission's Kirupananda Variyar Medical College and Hospital, Salem, IND; 2 Ophthalmology, Vinayaka Mission’s Kirupananda Variyar Medical College and Hospital, Salem, IND; 3 General Medicine, Vinayaka Mission’s Kirupananda Variyar Medical College and Hospital, Salem, IND; 4 Internal Medicine, Vinayaka Mission's Kirupananda Variyar Medical College and Hospital, Salem, IND

**Keywords:** india, diabetic retinopathy, diabetes, waist circumference, fasting blood sugar

## Abstract

Introduction

Diabetes mellitus (DM) has a long-term impact on retinal micro-blood vessels, culminating in the progression of diabetic retinopathy (DR); however; screening for DR is not widely used due to a lack of accessibility and economic constraints, especially in resource-limited settings. Thus, a longitudinal marker that is associated with the development of DR is required. This study sought to assess the association of DR with fasting blood sugar (FBS) levels and waist circumference.

Methodology

A cross-sectional study was conducted in a tertiary care hospital for one year. All individuals diagnosed with Type 2 DM (T2DM) and ≥45 years of age were included in the study. Individuals with fasting blood glucose levels of <126 mg/dl and <3 years of diagnosis with T2DM were excluded from the study. Individuals having one or more retinal microaneurysms or retinal blot hemorrhages, with or without any additional abnormalities, were diagnosed with DR. The results were analyzed using SPSS version 21 (IBM Corp., Armonk, NY).

Results

The prevalence of diabetic retinopathy among the study participants was 67.6 %. There was a significant association between increasing waist circumference (p = 0.009) and High FBS levels (p = 0.032) with the presence of DR.

Conclusion

Approximately two-thirds of the patients with T2DM aged >45 years and above have diabetic retinopathy. High FBS and waist circumference were associated with DR.

## Introduction

Diabetes mellitus (DM) is a metabolic condition that is caused by problems in insulin production, its action, or both. It is distinguished by chronic hyperglycemia and irregularities in carbohydrate, lipid, and protein metabolism [1]. Type 2 diabetes mellitus (T2DM) is the most prevalent form of diabetes, which accounts for 90% of all diabetics worldwide [1,2]. Individuals with T2DM are more likely to develop complications, such as cardiovascular disease (CVD), neuropathy, eye disease (retinopathy), and nephropathy. This retinopathy may lead to blindness [3,4]. DM has a long-term impact on retinal tiny blood vessels, culminating in the progression to retinopathy and visual impairment. Diabetic retinopathy (DR) is the leading cause of visual impairment in adults aged 20-65 years; one in three people with diabetes have some degree of DR and one in 10 develop a vision-threatening form of the illness. Globally, the number of persons with DR is expected to rise from 126.6 million in 2010 to 191.0 million by 2030, with the number of people with vision-threatening DR rising from 37.3 to 56.3 million if immediate action is not taken [5,6]. DR is asymptomatic until it has progressed to an advanced stage; however, it is mostly preventable with frequent ocular screenings and appropriate treatment before vision impairment. Gender, unregulated DM, chronic DM, the associated complication of nephropathy, and comorbidity with hypertension are risk factors for DR in diabetic patients [7-9]. However, screening for DR is not widely used due to a lack of accessibility and economic constraints, especially in resource-limited settings. Thus, a longitudinal marker that is associated with the development of DR is required. To date, few studies have assessed any association with DR. Therefore, this study was conducted to assess the association between DR, fasting blood sugar (FBS) levels, and waist circumference.

## Materials and methods

Study design and sampling

A cross-sectional study was conducted in a tertiary care hospital in Salem for a duration of one year (July 2021 to July 2022). Study participants were included using a convenient sampling technique.

Sample Size

Previous studies have shown that the prevalence of DR among diabetes is 5-7%. Thus, with a 7% prevalence and 5% absolute error, the minimum sample size needed for the current study with a 95 percent confidence interval is 120 (inclusive of a 10% non-response rate). The sample size was calculated by using the formula 3.86*p*q/d^2^, where, p is prevalence, q is a compliment of p and d is absolute precision (5%).

Ethical Clearance

This study obtained ethical approval from the Institutional Ethical Committee at Vinayaka Mission’s Kirupananda Variyar Medical College and Hospitals (VMKVMC&H), Salem (approval number VMKVMC&H/ICE/21/118).

Eligibility criteria

Inclusion criteria: After obtaining informed consent, all individuals who were recruited during the study period with the diagnosis of T2DM and were ≥45 years of age were included in the study. Exclusion criteria: Individuals with fasting blood glucose levels of <126 mg/dl; those diagnosed with terminally ill conditions; and those <3 years from their T2DM diagnosis were excluded from the study. Similarly, all patients who were diagnosed with impaired glucose tolerance, maturity-onset diabetes of the young (MODY), latent autoimmune diabetes in adults (LADA), and Type 1 DM were also excluded from the study.

Data collection

All study participants provided 12-hour overnight fasting blood samples, which were sent for laboratory analysis. The circumference line connecting the narrowest point between the inferior border of the lower rib, iliac crest, and umbilicus was used to measure the waist circumference. The normal waist circumference range for men and women is less than 90 and less than 80 centimeters respectively. Individuals presented with ≥1 retinal micro-aneurysm or retinal blot hemorrhages, with or without any additional abnormalities, while doing ophthalmoscopy, were diagnosed as DR.

Data analysis

The data obtained from the study were entered in Microsoft Excel. The results were analyzed using SPSS version 21 (IBM Corp., Armonk, NY). Continuous variables were expressed as mean and standard deviation. The association between FBS and waist circumference with DR was assessed by using an independent t-test. Data were considered statistically significant with a 95% confidence interval when p < 0.05.

## Results

This study included 213 individuals. The mean age, duration of diabetes, height, and weight were 60.46 years, 10.02 years, 161.24 cm, and 61.6 kg, respectively (Table 1).

**Table 1:** Distribution of the study participants according to their age and duration of diabetes mellitus (n = 213)

	Age in years	Duration of Diabetes	Height in Centimeters	Weight in Kilogram
Mean	60.46	10.02	161.24	61.61
Median	62.00	8.00	162.00	61.00
Mode	62	3	162	53
Std. Deviation	9.189	7.348	8.174	12.345
Minimum	40	3	142	33
Maximum	84	40	188	97
Interquartile range	53.5 - 66.0	156 - 167	54 - 68	4 - 15

The general characteristics of the study participants are described in Table 2. Approximately 55.9% of the study participants were males (n = 119). The prevalence of hypertension and being overweight was 13.6% and 24.9%, respectively. The prevalence of diabetic retinopathy among the study participants was 67.6%.

**Table 2:** General characteristics of the study participants (n = 213)

S. No	General characteristics		Frequency	Percent
1	Gender	Female	94	44.1
		Male	119	55.9
		Total	213	100.0
2	Diabetic retinopathy	No	69	32.4
		Yes	144	67.6
		Total	213	100.0
3	Hypertension	No	184	86.4
		Yes	29	13.6
		Total	213	100.0
4	High cholesterol	No	183	85.9
		Yes	30	14.1
		Total	213	100.0
5	Body Mass Index category	Underweight	16	7.5
		Normal	144	67.6
		Overweight	53	24.9
		Total	213	100.0

The Body Mass Index (BMI), waist circumference, and waist-hip ratio were shown in Table 3. The mean BMI among the study participants was 23.652. Similarly, the mean waist circumference was 39.04 inches.

**Table 3:** Distribution of the study participants according to their BMI, Waist circumference, and Waist hip ratio (n = 213)

		Body Mass Index	Waist circumference in Inch	Waist hip ratio
Mean		23.6526	39.04	.9236
Median		22.8624	38.00	.9184
Mode		23.62	34	.83
Std. Deviation		4.34557	5.452	.18682
Minimum		15.70	26	.10
Maximum		39.86	52	2.69
Percentiles	25	21.3599	35.00	.8799
	50	22.8624	38.00	.9184
	75	24.9880	43.50	.9444

Similarly, the mean fasting blood sugar (FBS) value, and systolic and diastolic blood pressure were 198.63 mg/dl, 132.70 mm Hg, and 76.81 mm Hg respectively (Table 4).

**Table 4:** Distribution of the study participants according to their FBS and blood pressure (n = 213) FBS: Fasting blood sugar

		Fasting Blood Sugar in mg/dl	Systolic Blood Pressure	Diastolic Blood Pressure
Mean		198.36	132.70	76.81
Median		204.00	130.00	80.00
Mode		229	130	70
Std. Deviation		49.276	21.921	10.778
Minimum		126	70	50
Maximum		323	190	120
Percentiles	25	151.00	120.00	70.00
	50	204.00	130.00	80.00
	75	229.00	145.00	80.00

The mean FBS value for participants with and without DR was 203.36 and 187.93, respectively (Table 5), which was a significant difference (p = 0.032). The mean waist circumference for participants with and without DR was 39.72 and 37.64, respectively, which was also a significant difference (p = 0.009).

**Table 5:** Association between FBS and waist circumference and diabetic retinopathy (n = 213)

Risk Factors	Diabetic retinopathy	N	Mean	Standard Deviation	Mean difference	P-Value
Fasting Blood Sugar in mg/dl	No	69	187.93	50.061	- 15.434	0.032
	Yes	144	203.36	48.274		
Waist circumference in Inch	No	69	37.64	5.785	- 2.078	0.009
	Yes	144	39.72	5.172		

## Discussion

This study found that the prevalence of DR among individuals with T2DM aged ≥45 years was 68%. A similarly high prevalence rate of 51% was found by Kaushik et al. in a hospital-based study conducted in a tertiary care center in Kashmir, India, in 2021 (N = 625) [10]. A hospital-based multicentric study performed across India by Rajalakshmi et al. in 2016 (N = 11,182) found a prevalence of 33% DR in the study population with a mean age of 58 years [11]. This difference may be due to sample size differences and a multi-centric approach. A population-based study (N = 63,000, age ≥50 years) involving 31 districts showed a prevalence of only 16.9% [12]. This study used data from a national survey from 2015-2019. This big difference could be due to differences in the study setting and sample size. Further research is required to confirm the true prevalence.

Our study showed a significant association between increased waist circumference and the presence of DR. Similar results have been reported in a Chinese population (N = 511) study [13]. That study assessed the association between central obesity (defined by waist circumference) and DR. Results indicated that individuals with central obesity are 1.07X more likely to have DR (95% confidence interval, 1.03-1.10). Further, Man et al. have shown that obesity, as defined by BMI, is protective against developing DR, whereas central obesity, as defined by waist circumference, is significantly associated with DR [14]. This indicates the importance of waist circumference among diabetic patients when considering the risk of developing DR. In contrast to our study, Wat et al. have reported no significant association between obesity and DR [15]. A review article by Mbata et al. has discussed a significant association between metabolic syndrome and the development of DR; however, there are conflicting data regarding the individual factors of metabolic syndrome on the development of DR, especially in non-diabetic individuals [16]. Taken together, these data highlight that central obesity/obesity among individuals with T2DM diabetics does not have a confirmed association with the presence of DR. Our study showed a significant association between increased waist circumference and DR; however, further evaluation is required.

Moreover, we found a significant association between high FBS and the presence of DR. Similarly, a study by Yin et al. has found a significant association between FBS and the prevalence of DR among individuals with diabetes [17]. Another cross-sectional study undertaken in China by Cui et al. found a similar association among 17,985 participants [18]. Further, Kumar et al. have shown that FBS and post-prandial blood sugar (PPBS) levels along with triglyceride levels are significantly associated with the prevalence of DR [19]. An indirect association was shown by Mujeeb et al. with 150 diabetes patients; FBS is associated with the urinary protein-creatine ratio, which in turn is associated with the prevalence of DR [20]. Therefore, our data add to the strength of the association between FBS and the presence of DR. 

Strengths and limitations of the study

This study follows a convenient sampling technique; however, a fair number of study participants were included in the study. All the data and measurements were collected by a single investigator, which eliminated any inter-observer bias. This study was performed in a tertiary care setting alone, which may cause Berksonian Bias. A multicentric or a community-based study would provide more reliable results when assessing the prevalence of DR.

## Conclusions

Approximately two-thirds of individuals with diabetes who were ≥45 years of age had DR in our study sample. High FBS levels and increased waist circumference were significantly associated with the presence of DR. Therefore, FBS and waist circumference may be useful tools to indicate further screening for DR, especially in low-resource settings.
